# Glutaminase inhibitors rejuvenate human skin via clearance of senescent cells: a study using a mouse/human chimeric model

**DOI:** 10.18632/aging.204391

**Published:** 2022-11-21

**Authors:** Kento Takaya, Tatsuyuki Ishii, Toru Asou, Kazuo Kishi

**Affiliations:** 1 Department of Plastic and Reconstructive Surgery, Keio University School of Medicine, Tokyo, Japan

**Keywords:** glutaminase inhibitor, human skin, senescent cell, aging, therapeutic agent

## Abstract

Skin aging caused by various endogenous and exogenous factors results in structural and functional changes to skin components. However, the role of senescent cells in skin aging has not been clarified. To elucidate the function of senescent cells in skin aging, we evaluated the effects of the glutaminase inhibitor BPTES (bis-2-(5-phenylacetamido-1, 3, 4-thiadiazol-2-yl)ethyl sulfide) on human senescent dermal fibroblasts and aged human skin. Here, primary human dermal fibroblasts (HDFs) were induced to senescence by long-term passaging, ionizing radiation, and treatment with doxorubicin, an anticancer drug. Cell viability of HDFs was assessed after BPTES treatment. A mouse/human chimeric model was created by subcutaneously transplanting whole skin grafts from aged humans into nude mice. The model was treated intraperitoneally with BPTES or vehicle for 30 days. Skin samples were collected and subjected to reverse transcription-quantitative polymerase chain reaction (RT-qPCR), western blotting, and histological analysis. BPTES selectively eliminated senescent dermal fibroblasts regardless of the method used to induce senescence; aged human skin grafts treated with BPTES exhibited increased collagen density, increased cell proliferation in the dermis, and decreased aging-related secretory phenotypes, such as matrix metalloprotease and interleukin. These effects were maintained in the grafts 1 month after termination of the treatment. In conclusion, selective removal of senescent dermal fibroblasts can improve the skin aging phenotype, indicating that BPTES may be an effective novel therapeutic agent for skin aging.

## INTRODUCTION

Cellular senescence is implicated in age-related tissue dysfunction and the subsequent development of age-related diseases [1]. Senescent cells, when present transiently, have a beneficial function in wound healing; however, their chronic accumulation adversely affects surrounding tissues via the senescence-associated secretory phenotype (SASP). Senescent cells with SASP increase the secretion of inflammation-inducing cytokines and chemokines, extracellular matrix remodeling proteases, and growth factors, consequently contributing to the gradual loss of tissue and organ function with aging [2–4].

“The senescent cells accumulate in the epidermis, dermis, and subcutaneous adipose tissue depots, and the skin contains approximately 15%–60% senescent fibroblasts [5]. The accumulation of senescent cells in the skin with aging has long been known [6, 7].” An emerging hypothesis of skin aging is that secretion of SASP by aging fibroblasts leads to extracellular matrix disruption and abnormal tissue remodeling, consequently altering the composition and structure of collagen and elastin fibers, inflammatory cell infiltration, and adipose tissue atrophy [8, 9]. In addition, aging-related stem cell depletion leads to a reduced response of skin tissue to damage and cell differentiation [10]. Skin aging include the characteristic aging-related aesthetic changes, including sagging, creases, color changes, skin thinning, atrophy, and degradation of the fat beneath the skin [11]. To antagonize these phenomena, senescent cells are emerging as new targets for novel therapies aimed at weakening the aging phenotype. Selective removal of senescent cells in transgenic animal models delays several age-related diseases and extends both healthy and overall life spans [12, 13]. In the skin, the removal of senescent cells can potentially eliminate the adverse effects of SASP by promoting cell differentiation and proliferation, normalizing the extracellular matrix, and reducing apparent inflammation. To date, several “senolytic drugs” have been developed to remove senescent cells. For instance, clearance of senescent cells in the skin after treatment with the senolytic drug cocktail dasatinib and quercetin reduced radiation ulceration [14]. Furthermore, studies using ABT-737, a pan-inhibitor of the BCL-2 family, showed selective removal of senescent cells in mice skin and 3D human epidermal equivalents [15, 16].

A recently reported senolytic drug is BPTES [bis-2-(5-phenylacetamido-1,3,4-thiadiazol-2-yl)ethyl sulfide], a glutaminase 1 (GLS1) inhibitor [17]—GLS1 is an essential factor for the survival of human senescent cells in living tissues, which should normally be eliminated by apoptosis. In senescent cells, apoptosis is evaded by induction of kidney-type glutaminase (KGA) expression in response to a decrease in intracellular pH due to lysosomal membrane damage, which promotes glutaminolysis [18]. Inhibition of KGA-dependent glutaminolysis in old mice specifically eliminates senescent cells, improves age-related organ dysfunction, and promotes wound healing in the skin [17]. However, the effect of BPTES on the aging skin phenotype remains unclear.

In addition, several limitations exist in the *in vivo* models commonly used in the field of skin aging. There are fundamental differences between rodent and human skin structures that limit their use in studying effects on the skin. Furthermore, significant differences exist in the factors involved in melanocyte layer structure [19], hair cycle [20], immune system, cell signaling, oncogenes, and tumor suppression [21, 22]. Mammalian skin, such as that of pigs, resembles human skin but is more costly [23]. Moreover, 3D human skin equivalents, an alternative to animal models, are technically very complex and cannot be used for long-term follow-up. Moreover, their structural complexity and cellular diversity are inferior compared to the skin *in vivo* [24]. Therefore, we utilized plastic surgery to create an experimental mouse/human chimeric model in which intraoperatively obtained human whole skin layers were transplanted into nude mice using previously described methods [25] and evaluated the anti-aging effects of BPTES on real human skin.

## RESULTS

### Increased expression of aged markers in senescent dermal fibroblasts

**Figure 1:**
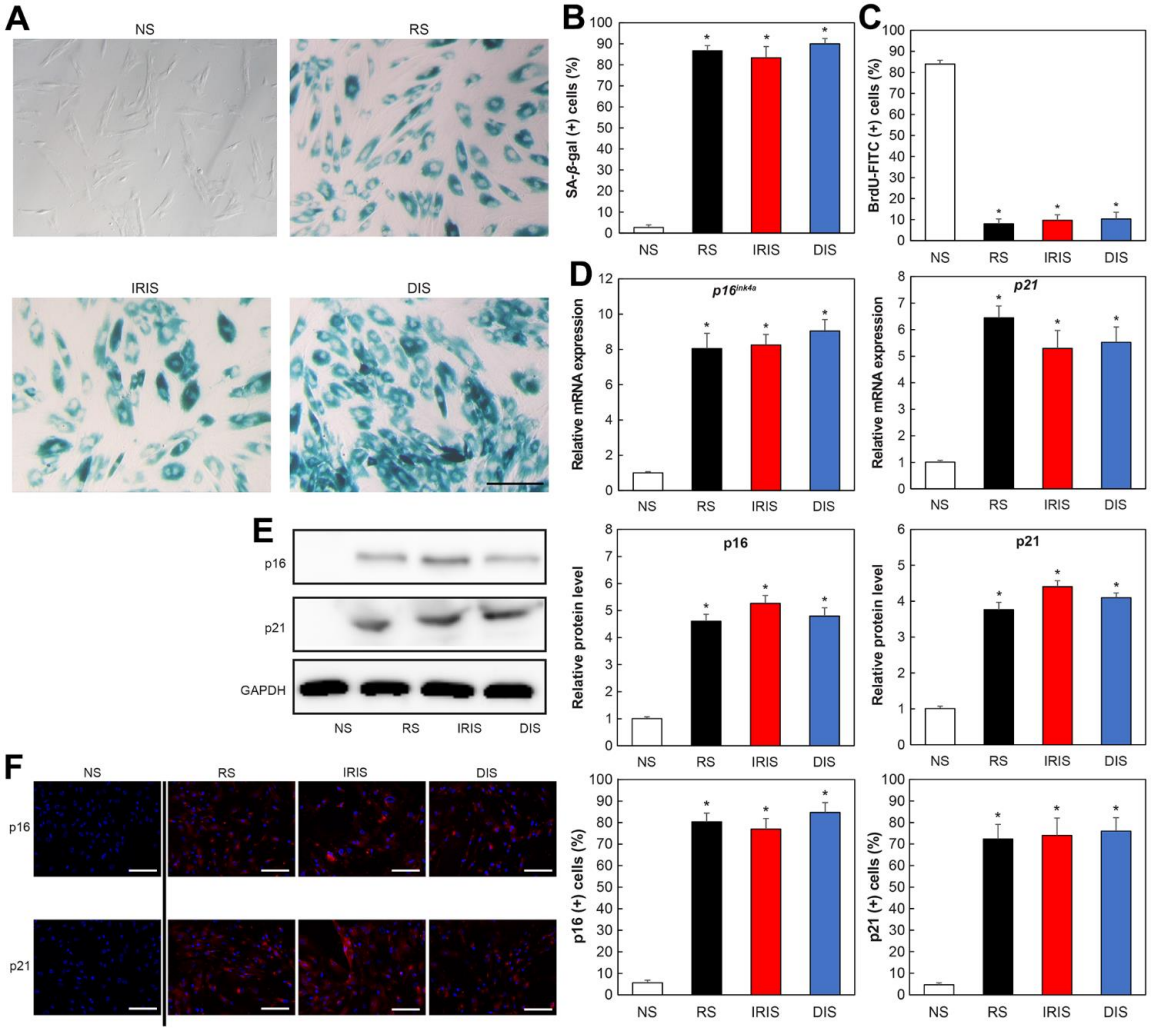
**Increased aging-related markers in aged HDFs.** (**A**) SA-β-Gal-stained image. Bar = 50 μm. (**B**) Percentage of SA-β-Gal-positive cells. (**C**) Percentage of BrdU-FITC-positive cells determined using flow cytometry. (**D**) Comparison of relative gene expression of *p16* and *p21*. *p < 0.05. (**E**) Comparison of protein expression of p16 and p21. (**F**) Immunostaining of p16 and p21. Bar = 50 μm. All results are expressed as mean ± SEM. All experiments were independently repeated in triplicate. NS: non-senescence.

As a model of senescence in HDFs, we used replicative senescence (RS) induced by more than 45 cell passages, ionizing radiation-induced senescence (IRIS) induced by irradiating cells with ionizing radiation, and doxorubicin-induced senescence (DIS) induced by treating cells with doxorubicin. Senescence-associated β-galactosidase (SA-β-Gal) assay confirmed the senescence of the cells with each treatment. The cytoplasm of senescent fibroblasts showed a flattened and expanded morphology and increased SA-β-Gal activity (Figure 1A). Positive cells were significantly increased compared to non-senescence cells (NS) in all aging models (Figure 1B; RS: *p* = 0.0000072, IRIS: *p* = 0.0046, DIS: *p* = 0.000072). Furthermore, the Bromodeoxyuridine (BrdU) assay used to assess the cell division activity revealed lower activity in senescent cells than that in NS (Figure 1C; RS: *p* = 0.000014, IRIS: *p* = 0.00016, DIS: *p* = 0.00026).

The expression of aged markers such as p16 and p21 was also analyzed using real-time polymerase chain reaction (PCR). The mRNA expression of *p16* and *p21* in senescent cells was increased compared with that in NS cells (Figure 1D; p16: RS: *p* = 0.000012, IRIS: *p* = 0.000028, DIS: *p* = 0.000019; p21: RS: *p* = 0.00022, IRIS: *p* = 0.000044, DIS: *p* = 0.00021). Western blotting also showed that p16 and p21 proteins were upregulated in senescent cells compared with that in NS cells (Figure 1E; p16: RS: *p* = 0.0053, IRIS: *p* = 0.0048, DIS: *p* = 0.0067; p21: RS: *p* = 0.0060, IRIS: *p* = 0.00031, DIS: *p* = 0.00023).

Furthermore, immunofluorescence staining of p21 and p16 expression revealed that the numbers of p21-positive (RS: *p* = 0.0033, IRIS: *p* = 0.005, DIS: *p* = 0.0036) and p16-positive (RS: *p* = 0.01, IRIS: *p* = 0.013, DIS: *p* = 0.0077) cells were elevated in the three senescent cell models (Figure 1F). These results confirm that cellular senescence is appropriately induced in dermal fibroblasts in the three models.

### BPTES selectively eliminates human skin senescent fibroblasts

**Figure 2:**
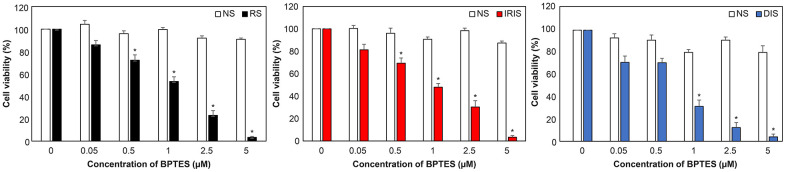
**Selective removal of senescent HDFs by treatment with BPTES.** After induction of cellular senescence, cells were treated with BPTES at the indicated concentrations for 72 h (n = 4). Cell viability was measured to determine the effect of the senescent cell-removal drugs. Histograms were compared to the DMSO control. The graph shows the mean ± SEM of three independent experiments; *p < 0.05, Mann–Whitney *U test;* NS: non-senescence.

To selectively eliminate senescent skin fibroblasts *in vitro*, multiple concentrations of BPTES were administered to NS and senescent cells to determine its efficacy. NS cell viability was > 80% at all treatment doses (white bars), while senescent cell viability was more than that of control (dimethyl sulfoxide [DMSO] treatment). Cell viability of RS, IRIS, and DIS were all significantly decreased after BPTES treatment, with RS and IRIS ranging from 0.5 to 5.0 μM and DIS from 1.0 to 5.0 μM (Figure 2). These data show that BPTES selectively kills HDFs *in vitro*.

### BPTES reduces SA-β-Gal-positive cells in the skin of aging humans in the chimeric model

**Figure 3:**
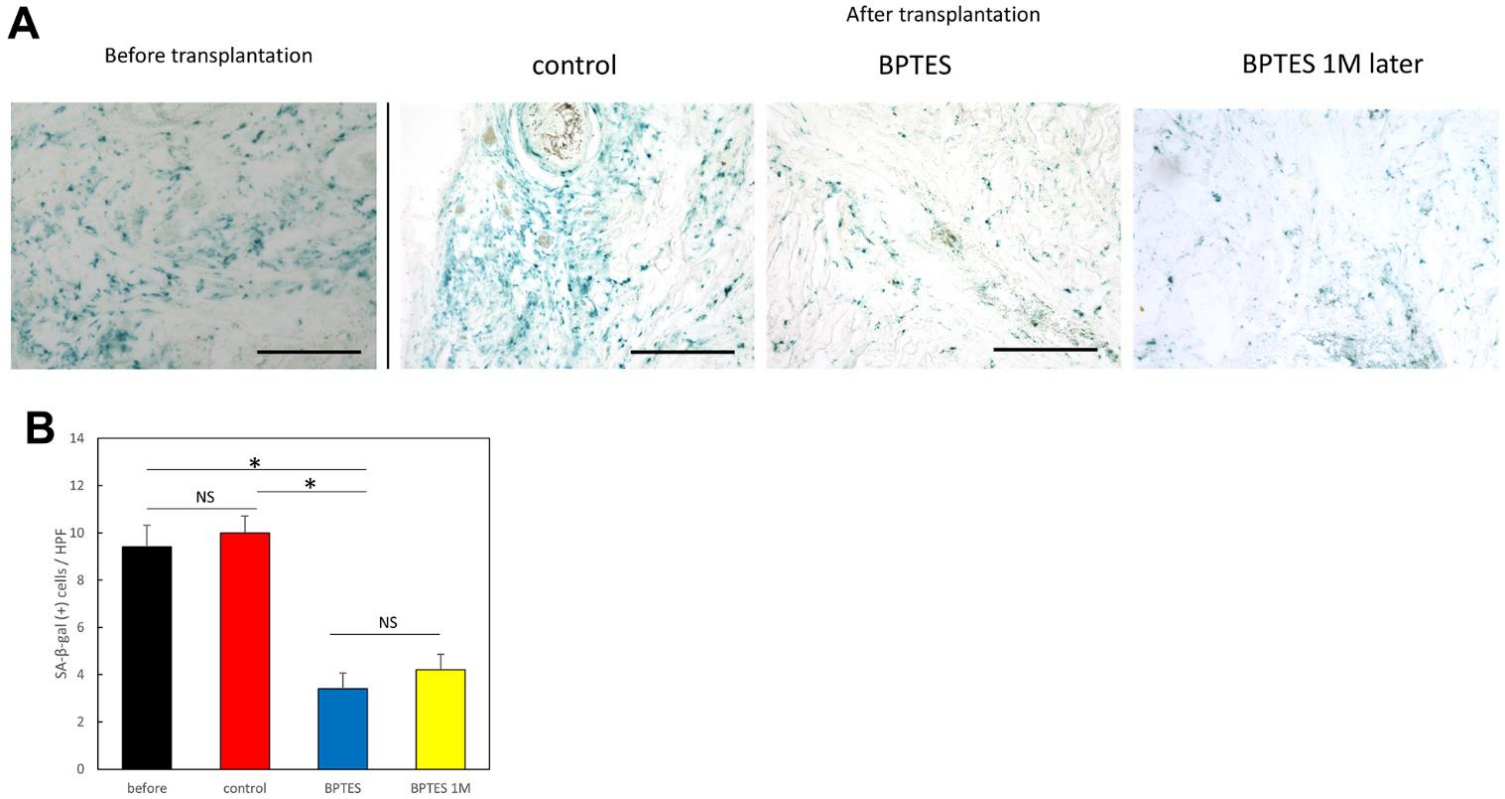
**Removal of SA-β-Gal-positive cells from human skin grafts by BPTES treatment.** (**A**) Skin tissue stained with SA-β-Gal (blue); Bar = 100 μm. (**B**) Quantification of SA-β-Gal-positive cells in the dermis, excluding the area of the skin appendage. The number of positive cells was counted in high magnification field of view (HPF) (≥5 fields of view per sample). Graphs show the mean ± SEM of three independent experiments. *p < 0.05.

To investigate the effects of BPTES on aging skin *in vivo*, we used a mouse/human chimeric model. Specifically, we transplanted aged human skin grafts into nude mice and observed the effects of BPTES treatment on the grafts. First, frozen sections of the transplanted skin grafts were stained with SA-β-Gal (Figure 3A). SA-β-Gal-positive signals in the dermis were considered to indicate aging dermal cells. The number of SA-β-Gal positive cells among all cells counted in the high magnification field of view was significantly reduced by BPTES treatment (Figure 3B; before vs. BPTES: *p* = 0.0012; control vs. BPTES: *p* = 0.00015). In contrast, the number of SA-β-Gal-positive cells did not change significantly in the control treated with the vehicle compared with that in the pre-transplant group (*p* = 0.62). There was also no regression in the number of SA-β-Gal-positive cells in skin grafts 1 month after the administration of the last dose (BPTES vs. BPTES 1M later: *p* = 0.56). These results indicate that the senescent cell scavenger BPTES can eliminate SA-β-Gal-positive cells in a human skin graft chimera model.

### BPTES-induced decrease in p16 expression and increase in Ki67 expression in aged human skin grafts

**Figure 4:**
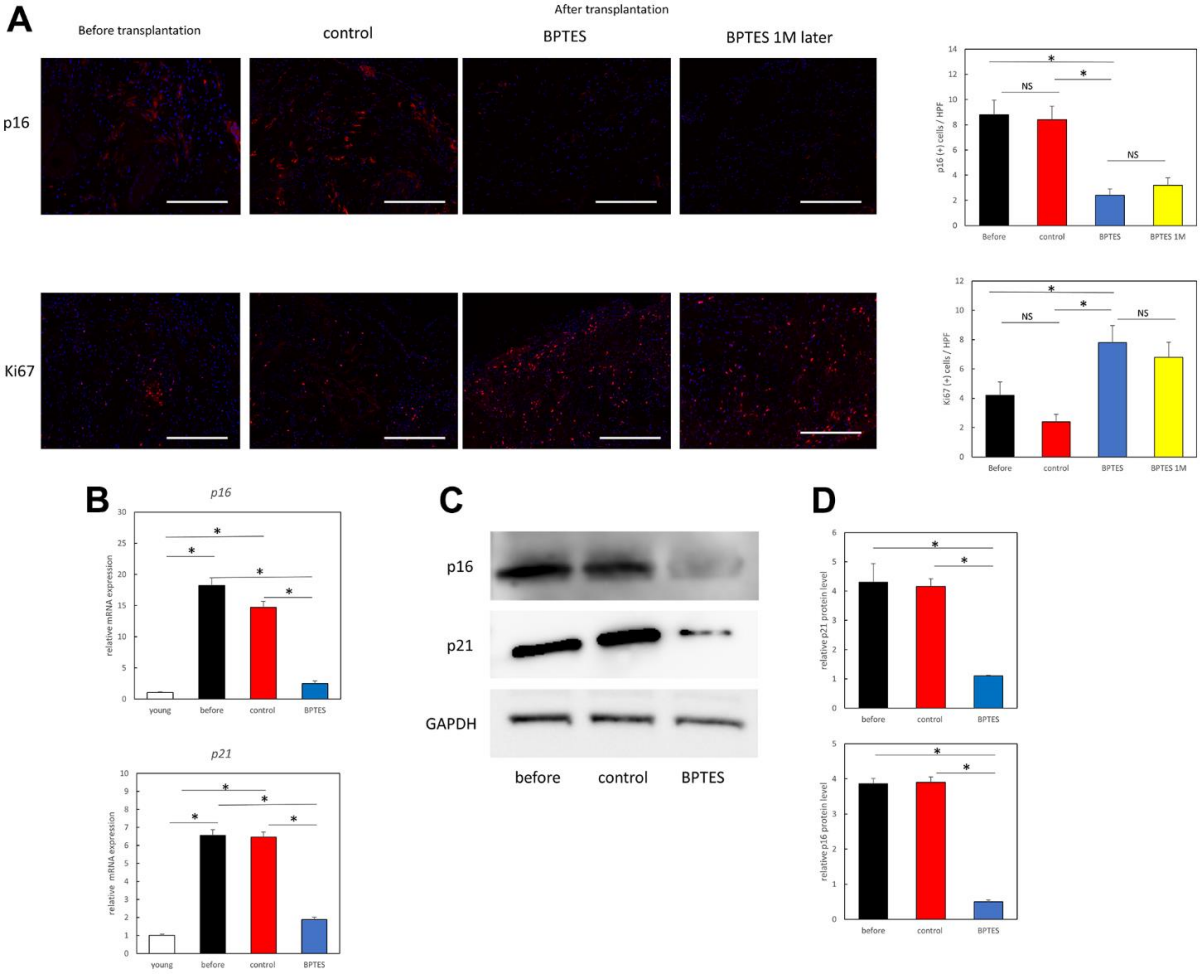
**BPTES treatment promotes the removal of skin senescent cells and cell proliferation from human skin grafts.** (**A**) Immunofluorescence staining for p16 or Ki67 in tissue sections of human skin grafts from pre-transplanted aged human skin, treated with control (DMSO) or BPTES. Red: p16, Ki67; Blue: DAPI. The percentage of p16- or Ki67-positive dermal cells at a depth of 100 μm from the epidermal basement layer was counted with DAPI. All results are expressed as mean ± SEM. *p < 0.05. Bar = 100 μm. (**B**) Relative p16 and p21 mRNA levels were analyzed using RT-qPCR in skin sections of the indicated mice. *p < 0.05. (**C**) Western blot analysis of p16 and p21. (**D**) Quantitative analysis of western blot. Band densities normalized against endogenous control GAPDH are shown. *p < 0.05. Graph shows mean ± SEM of three independent experiments.

To further confirm whether the p16-positive aging fibroblasts seen *in vitro* were eliminated from the dermis, we estimated p16 expression in the transplanted skin grafts using immunostaining. A large number of p16-positive cells were found in the dermis of aging skin before transplantation and control skin fragments, while they were significantly reduced in skin treated with BPTES. This effect was maintained till 1 month after the administration of the last dose (Figure 4A; before vs. BPTES: *p* = 0.0039; control vs. BPTES: *p* = 0.0024; BPTES vs. BPTES 1M: *p* = 0.61).

No significant changes were observed between the pre-transplanted skin treated with the DMSO and the skin before transplantation (*p* = 0.81). Furthermore, to assess cell proliferation related to skin aging, we estimated the expression of Ki67, which identified a large number of Ki67-positive cells in the dermis of BPTES-treated skin fragments. The increased number of Ki67-positive cells was also observed 1 month after the end of treatment (Figure 4A; before vs. BPTES: *p* = 0.04; control vs. BPTES: *p* = 0.0076; BPTES vs. BPTES 1M: *p* = 0.62). No significant difference was observed between before-treatment and DMSO-treated skin grafts (*p* = 0.14).

In addition, real-time PCR showed that the aging-related markers *p16* (before vs. BPTES: *p* = 0.0066; control vs. BPTES: *p* = 0.0024) and *p21* (before vs. BPTES: *p* = 0.0013; control vs. BPTES: *p* = 0.0013) were significantly suppressed at the mRNA level in the BPTES-treated group compared with that in the pre-transplant and control-treated groups (Figure 4B). Western blot analysis showed a significant accumulation of p16 and p21 in the skin before transplantation and in the control-treated group; however, their levels were significantly reduced in the BPTES-treated group (Figure 4C). Quantitative analysis demonstrated that relative p16 protein levels were significantly reduced in BPTES-treated skin grafts compared with that in pre-transplant and control-treated skin grafts (Figure 4D; before vs. BPTES: *p* = 0.0013; control vs. BPTES: *p* = 0.0013). Moreover, relative p21 protein levels were also significantly reduced in BPTES-treated skin grafts compared with that in pre-implantation and control-treated skin grafts (Figure 4D; before vs. BPTES: *p* = 0.0013; control vs. BPTES: *p* = 0.0013). These results suggest that treatment with senescent cell-depleting drugs may reduce senescent cells in the skin of aging mice.

### BPTES attenuates SASP expression in aging human skin

**Figure 5:**
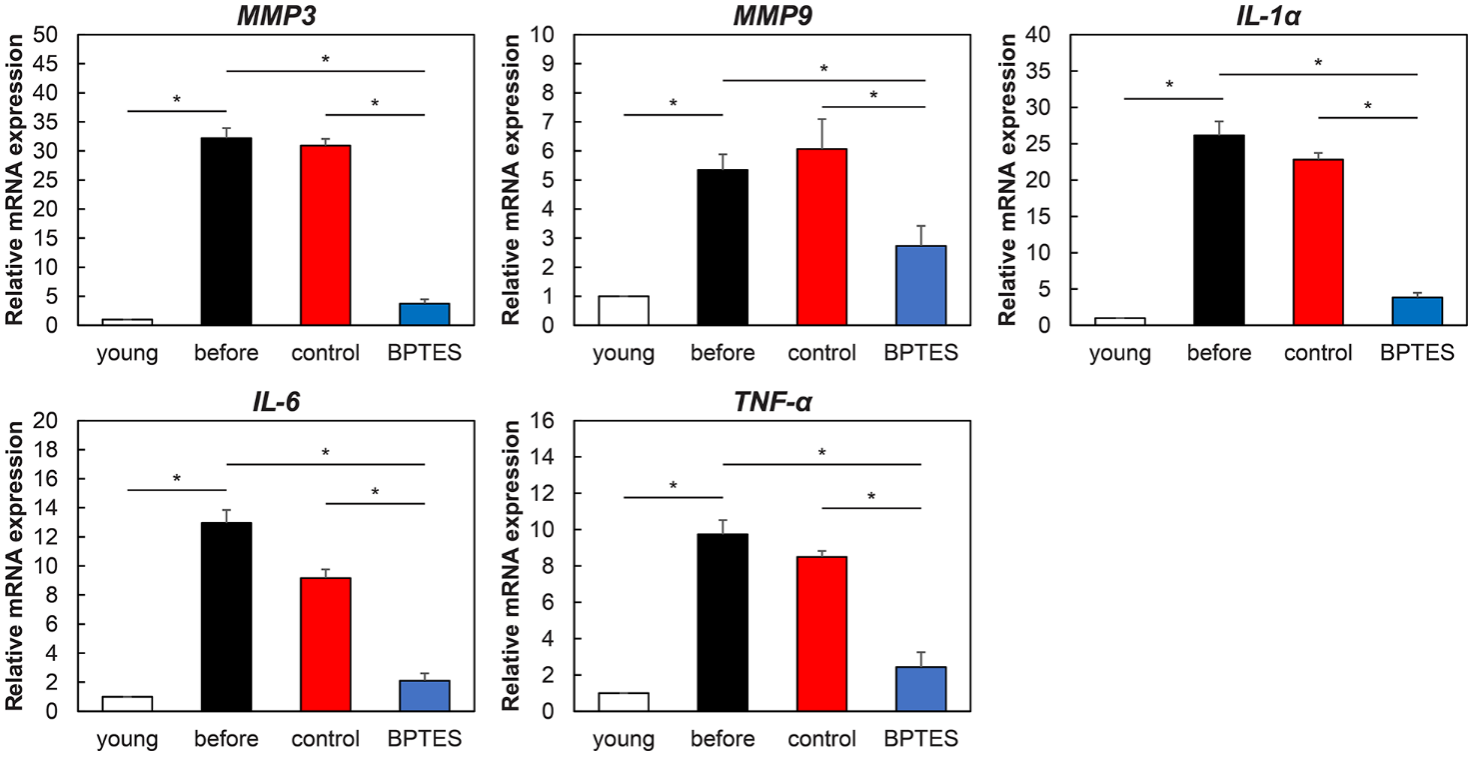
**Effect of senescent cell-depleting agents on SASP expression in aged human skin grafts.** Relative mRNA levels of genes associated with SASP in mouse skin measured using qRT-PCR. Data were normalized against mRNA obtained from young human skin; *GAPDH* was used as endogenous control. All results are expressed as mean ± SEM. *p < 0.05.

Next, we investigated the effect of BPTES on SASP in aged human skin grafts. RT-qPCR revealed that mRNA expression of SASP molecules, such as *Mmp-1*, *Mmp-3*, *Il-1a*, *IL6*, and *TNFα*, was elevated in aged skin. The expression of these SASP molecules exhibited no significant change in the control-treated group, whereas BPTES treatment reduced their expression (Figure 5). Therefore, we inferred that endogenous aging may be associated with elevated SASP and that the application of senescent cells may weaken SASP secretion by removing senescent cells from aging human skin.

### BPTES improves the phenotype of aging skin

**Figure 6:**
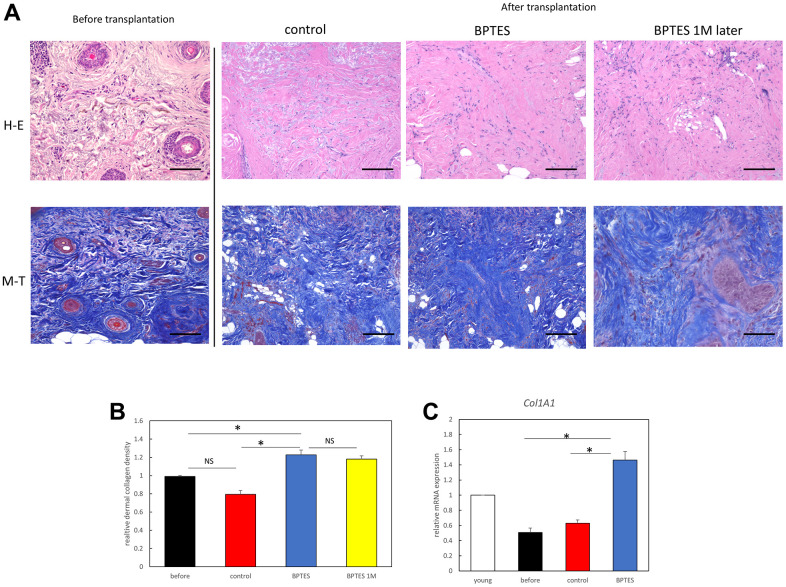
**Senescent cell elimination drug BPTES improves the skin senescence phenotype of transplanted aged human skin.** (**A**) Evaluation of skin collagen deposition using representative images of MT and H&E staining. (**B**) Quantification of collagen density in skin sections from the indicated mice. Data represent three or more random sites in each section (n = 3–5). (**C**) Relative mRNA levels of *Col1a1* in the skin from aged mice. *p < 0.05, Mann–Whitney *U* test; bar = 100 μm.

Next, human skin transplanted into nude mice was analyzed histologically. To evaluate the efficacy of senescent cell-depleting agents on skin aging, hematoxylin and eosin (H&E) staining and Masson’s trichrome (MT) staining were performed to evaluate morphological changes in human skin grafts and collagen fiber density in the dermis. Inflammatory cell infiltration was observed in the dermis in the transplant group. Collagen density was significantly reduced in aged skin, but density was improved with BPTES treatment and was maintained in the tissue grafts 1 month after treatment (Figure 6A, 6B). Consistent with MT data, *Col1a1* mRNA expression was decreased in the pre-transplanted and the control-treated skin graft but was restored in the BPTES-treated skin graft (Figure 6C).

## DISCUSSION

The skin is constantly at risk of damage as it is a regenerative organ in which certain structures, such as the epidermis and hair follicles, exhibit growth activity and serves as the outermost protective layer of the body. It has been confirmed that aging exacerbates this damage and increases the number of senescent cells within the skin in an age-dependent manner [26]. Based on this evidence of cellular senescence and its correlation with both aging and age-related diseases, senescent cell accumulation has been postulated as causally related to age-related skin dysfunction and phenotype [27, 28].

This study aimed to determine the role of senescent fibroblasts in endogenous skin aging by eliminating senescent dermal cells using the senescent cell eliminator BPTES. First, to evaluate the effects of known senescent cell eliminators on skin cells, three types of cellular senescence were induced in primary human skin fibroblasts: (i) RS, (ii) IRIS, and (iii) DIS. BPTES was effective in removing senescent cells in all skin fibroblast models. To evaluate its effects on human skin, nude mice were transplanted with whole human skin and treated with BPTES, which improved dermal collagen density, accelerated cell division, and SASP secretion. In summary, our results indicate that BPTES application may induce selective removal of senescent cells and suppression of skin aging-related phenotypes.

Inflammation is considered a major cause of aging and age-related diseases [29]. Human fibroblasts isolated from aging skin express secreted proteins associated with inflammation, known as SAASPs [30]. SAASPs include interleukins (IL-6, IL-8, and IL-1β), MMPs (1, 3, 10, and 14), and TNFα, which are closely associated with skin-specific matrix degradation and inflammation-inducing processes. In particular, MMPs, such as MMP-1, MMP-3, and MMP-9, have been described to increase with age and promote the skin aging phenotype [31]. This study demonstrated that DNA damage was induced in senescent cells via replicative senescence, radiation, and anticancer drug administration. These damages were selectively cleared by BPTES, which suppressed several SASP factors that were increased in the aged human skin *in vivo*. These findings indicate that senescent cell eliminators improve the skin aging phenotype by suppressing SASP associated with skin aging via selective elimination of senescent fibroblasts.

Human skin is an ideal preclinical aging research model but is seldom used in mainstream aging research for this purpose [31, 32]. We have previously shown that transplantation of aged human skin into immunocompromised young mice restores several aging-related parameters in the epidermis of human xenografts [33, 34]. However, it was unclear whether the skin rejuvenation effects observed in these studies extended beyond the epidermis. Recent studies have identified vascular endothelial growth factor (VEGF) as a major driver of human organ rejuvenation *in vivo* [35]. Specifically, VEGF-A in young mice has been reported to rejuvenate the aging skin phenotype via enhanced angiogenesis in human xenodermal grafts [36]. This study revealed no obvious rejuvenation effect in the control-treated group after transplantation. In contrast, rejuvenation of transplanted skin was improved in the BPTES-treated group via clearance of senescent cells and SASP suppression, compared to that of the pre-transplanted or control-treated groups. The first report did not target the clearance of dermal fibroblasts [17], and this report has the potential to the development of skin rejuvenation therapies.

However, our study has certain limitations. The skin samples used in this study were collected from male volunteers only; therefore, the effect of BPTES on female skin is unknown. Furthermore, due to sample size limitations, it is unclear in which age group this rejuvenation effect would be useful. Therefore, this chimeric model should be tested in the future to validate the role of BPTES in a larger number of human skin samples. In addition, it is difficult to observe macroscopic changes that occur in the skin in this chimeric model. In this study, the skin fragments were implanted subcutaneously in mice to prevent self-injury and shedding, which caused these fragments to shrink and modify the surface more than their original state. Therefore, experimental models need to be established to determine the effect of BPTES on the surface properties of human skin. Furthermore, no adverse events associated with BPTES could be identified under the present study conditions. However, activated T cells are known to utilize glutaminolysis for proliferation, and inhibiting this activity may interfere with the innate immune response to new tumor formation [37]. Especially because many metabolic pathways involved in cancer cell survival and invasion are shared with normally activated T lymphocytes [38], its long-term side effects, including tumorigenesis, should be explored in a future study.

In summary, our results indicate that selective clearance of aging dermal fibroblasts by BPTES ameliorates skin senescence-related changes and that aging dermal fibroblasts may play an important role in the skin aging process. Therefore, senescent cell eliminators for aging skin cells may be an effective option for treating skin aging.

## MATERIALS AND METHODS

### Cell culture

Normal HDFs (C-12300) were purchased from PromoCell GmbH (Heidelberg, Germany). HDFs were cultured on low glucose Dulbecco’s Modified Eagle’s medium (DMEM; Wako Pure Chemicals, Osaka, Japan) supplemented with 10% fetal bovine serum (Thermo Fisher Scientific, Waltham, MA, USA) and 1% penicillin/streptomycin (Wako Pure Chemicals). The cell cultures were maintained in a humidified incubator at 5% CO_2_ and 37° C.

### Induction of senescence to HDF

RS was induced by long-term passaging. Cells were divided every 2–3 days at a 1:3 ratio according to their proliferation rate and defined as RS when they reached a population doubling level (PDL) of 45 or higher. For IRIS, cells were exposed to 10 Gy of X-rays using CellRad (Faxitron, Tucson, AZ, USA) and collected after 10 days. DIS was defined as cell aging after treatment with 0.1 μM doxorubicin (Sigma-Aldrich, St. Louis, MO, USA) twice, followed by analysis 7 days of culture.

### Senescence-associated β-galactosidase staining

SA-β-Gal staining was performed using a kit (Senescence β-Galactosidase Staining Kit #9860, Cell Signaling Technology, Inc, Danvers, MA, USA) according to the manufacturer’s protocol. Briefly, cells were fixed in acetone at 20–25° C for 15 min, washed with phosphate-buffered saline (PBS) and then incubated overnight with SA-β-Gal staining solution (X-gal, 1 mg/mL; sodium citrate/sodium phosphate, pH 5.8, 40 mmol/L; potassium ferricyanide, 5 mmol/L; potassium ferricyanide, 5 mmol/L; NaCl, 150 mmol/L; MgCl2, 2 mmol/L) at 37° C. SA-β-Gal positive cells were visually counted in at least three microscopic fields of view in each sample.

For SA-β-Gal staining of frozen skin sections, tissues were embedded in an OCT compound (Sakura Finetek, Tokyo, Japan), sliced into 6 μm thick sections, and rehydrated in PBS. Afterward, the sections were fixed in acetone for 2 min and incubated overnight at 37° C with SA-β-Gal staining solution. After washing with PBS, bright-field images were collected using an upright microscope (NIKON ECLIPSE Ci-L; Nikon Instruments Inc., Melville, NY, USA) and analyzed using the image software ImageJ (Ver. 1.53p, National Institutes of Health, Bethesda, MD, USA). At least three random images were used in each sample acquired at 400x magnification. Blue signals in the dermis, excluding the epidermis and skin appendages, were counted as positive cells.

### Assessment of BrdU incorporation in fibroblasts using flow cytometry

To measure cell proliferation, cells were processed using the BrdUFlowEx FITC Kit (EXBIO Praha, a.s., Vestec, Czech Republic) according to the manufacturer’s protocol. A flow cytometer (BD Biosciences, Franklin Lakes, NJ, USA) and FlowJo (version 10.2) were used for analysis.

### Immunocytochemistry

Cells were placed on glass slides, fixed in 4% paraformaldehyde at 20–25° C for 10 min, and washed three times with PBS before staining. Afterward, the cells were incubated overnight at 4° C with primary antibodies [anti-p16ink4a antibody (ab211542; Abcam, Cambridge, UK) or anti-p21 antibody (ab109520; Abcam) diluted 1:100 in PBS]. Subsequently, the slides were washed three times in PBS, stained with AlexaFluor 555-conjugated anti-goat or anti-rabbit antibody (Thermo Fisher Scientific), and incubated for 1 h at 20–25° C. Then the slides were washed three times with PBS, and nuclei were counterstained and visualized using ProLong Gold Anti-fade Mountant (Thermo Fisher Scientific) containing 4′,6-diamidino-2-phenylindole (DAPI).

### Drug treatment and cell viability assays

Aged or normal human skin fibroblasts were plated in 96-well plates (5 × 10^3^ cells per well, *n* = 4) and maintained in 100 μL medium. Twenty-four hours later, the indicated concentrations of BPTES (0.05, 0.5, 1, 2.5, and 5 μM) or DMSO control were added to each well, respectively. BPTES was purchased from AdipoGen Life Sciences, Inc. (San Diego, CA, USA). After 72 h of incubation, cell viability was determined using the CellTiter-Glo^®^ 2.0 Cell Viability Assay (Promega, Madison, WI, USA). Relative viability was normalized against the DMSO control, and quantification experiments were performed in triplicate.

### Mouse/human chimeric model

Five-week-old male nude mice were purchased from Sankyo Laboratory Services (Tokyo, Japan). Mice were housed in ventilated cages in temperature-controlled rooms (21 ± 1° C) with a 12 h light/12 h dark cycle. Food and water were freely available.

Human skin grafts were collected from the trunks of five healthy male volunteers (ages 78–91 years, mean 84.5 years). Only males were selected to avoid hormonal effects. The volunteers had no obvious underlying diseases, no history of internal medicine, and no skin lesions were present on the skin samples. Mice were anesthetized using 3% isoflurane inhalation anesthesia. The dorsal portion of the mouse was first incised, and the subcutaneous area was peeled off with surgical scissors slightly larger than the graft. The skin grafts were processed into 1 cm × 1 cm squares and placed under the skin of the mice. The wound was tightly sutured with 4-0 nylon. Visual inspection of the grafts was performed daily. Starting 1 month after transplantation, BPTES (0.25 mg/20 g/200 μL in DMSO) or vehicle control (200 μL 10% DMSO in corn oil) was administered intraperitoneally 2–3 times a week for 1 month, after which the graft was retrieved. In addition, grafts were also collected 1 month after the end of the administration and analyzed.

### RNA extraction and reverse transcription

Total RNA was extracted from cells using a monophasic solution of phenol and guanidine isothiocyanate (ISOGEN; Nippon Gene, Tokyo, Japan) according to the manufacturer’s instructions. Total RNA was mixed with random primer, reverse transcriptase, and deoxynucleotide mixture (Takara Bio Inc., Tokyo, Japan). The mixture was incubated in a T100™ thermal cycler (Bio-Rad Laboratories, Inc., Hercules, CA, USA) at 25° C for 5 min annealing, 55° C for 10 min synthesis, and 80° C for 10 min heat inactivation of reverse transcriptase to obtain cDNA.

### Real-time quantitative polymerase chain reaction (RT-qPCR)

RT-qPCR was performed using cDNA and a TaqMan Fast Advanced Master Mix (Thermo Fisher Scientific) on an Applied Biosystems 7500 Fast Real-Time PCR System (Thermo Fisher Scientific). PCR was performed in 40 cycles of denaturation at 95° C for 3 sec and extension and annealing at 60° C for 30 sec. Subsequently, during the melting curve analysis phase, the temperature was increased from 60° C to 95° C, and fluorescence was measured continuously. The following probes were used for gene expression: MMP3 (assay ID. Hs00968305_m1), MMP9 (Hs00957562_m1), Il-6 (Hs00985639_m1), Il-1a (Hs00174092_m1), p16ink4a (Hs00923894_m1) and TNFα (Hs00174128_m1), Col1a1 (Hs00164004_m1) (all from Thermo Fisher Scientific). GAPDH (Hs02786624_g1) was used as an endogenous control. For *in vitro* analysis, *GAPDH* expression in the proliferating cell population was used as the baseline; for *in vivo* analysis, cDNA purified from mRNA previously collected from the normal human skin of a 10-year-old male was used as the relative baseline [39].

### Western blotting

Total proteins were extracted from cells and tissues in radioimmunoprecipitation assay buffer (Wako Pure Chemicals, Osaka, Japan). Each sample (40 μg) was electrophoresed on 10% polyacrylamide Mini-PROTEAN® TGX™ Precast Gels (Bio-Rad Laboratories, Inc.) at 200 V, 50 mA. The cells were then transferred to polyvinylidene difluoride membranes (Millipore, Bedford, MA, USA) using the Trans-Blot Turbo Transfer System (Bio-Rad Laboratories, Inc.). The membranes were blocked with 3% nonfat milk for 1 h at room temperature (20–25° C) and incubated overnight at 4° C with primary antibodies [p16 (ab108349, Abcam; 1:200), p21 (ab220206, Abcam; 1:100), GAPDH (Santa Cruz Biotechnology, Santa Cruz California, USA; 1:2,000)] diluted in blocking solution. After three times washing with TBST, the cells were incubated with secondary antibodies [donkey anti-goat IgG H&L (HRP) (ab6885; Abcam), goat anti-rabbit IgG H&L (HRP) (ab205718; Abcam), and goat anti-mouse IgG H&L (HRP) (ab205719; Abcam) (1:1000 dilution)] for 1 h at 20–25° C. Subsequently, the cells were washed three times with TBST and protein bands were visualized using an electrochemiluminescence detection kit (Pierce Biotechnology, Rockford, IL, USA). Images of the bands were acquired using a chemiluminescence imager (ImageQuant LAS4000mini, GE Healthcare, Chicago, IL, USA) and analyzed using ImageJ software. Each experiment was repeated three times.

### Immunohistochemistry

Frozen specimens were sliced into 7 μm-thick sections, mounted on glass slides, and fixed in acetone for 10 min at room temperature (20–25° C). To block nonspecific binding sites, the slides were incubated with 2% goat serum in PBS for 30 min at room temperature (20–25° C). Slides were then incubated overnight at 4° C with the following primary antibodies: p16 (ab108349, Abcam, 1:200) and Ki67 (ab16667, Abcam, 1:200). After washing three times with PBS, slides were incubated at room temperature (20–25° C) with Alexa Fluor 488-labeled goat anti-rabbit antibody and Alexa Fluor 555-conjugated goat anti-mouse antibody (both from Thermo Fisher Scientific), diluted 1:1000 in PBS for 1 h. After washing three times with PBS, the samples were counterstained for nuclear visualization using ProLong Gold Anti-fade Mountant (Thermo Fisher Scientific) containing DAPI.

### Masson’s trichrome and hematoxylin and eosin staining

Paraffin-embedded specimens were sliced into 7 μm-thick sections and mounted on glass slides. After drying overnight at room temperature (20–25° C), the slides were deparaffinized by 5 xylene exchanges (3 min each). Slides were transferred twice to 100% ethanol (2 min each) and then once to 95%, 80%, and 70% ethanol and water (3 min each) at room temperature (20–25° C) and rehydrated; MT staining and H&E staining were performed. For quantification of collagen density, images were captured in image J, the background of MT images was subtracted (n = 3 to 5, at least 3 microscopic fields per group), and RGB images were separated into 3 8-bit background images of each color using intrinsic software. To quantify collagen intensity, the blue component was evaluated, and only the region of interest (ROI) was measured. In brief, a rectangular ROI (0.01 mm^2^) was randomly assigned to the area under the basement membrane other than the skin appendages; the accumulation density was measured as the sum of pixel values within the selected ROI.

### Statistical analysis

All statistical analyses were performed using Statistica software version 9.0 (StatSoft, Tulsa, OK, USA). Data are expressed as mean ± standard error of the mean. The differences between the two groups were estimated using the Mann-Whitney U test. One-way ANOVA followed by Tukey’s post hoc test was used to compare differences between three or more groups. A p-value of < 0.05 was considered statistically significant.

### Data availability statement

The data that support the findings of this study are available from the corresponding author, K.T., upon reasonable request.
